# Experiences and emotional strain of NHS frontline workers during the peak of the COVID-19 pandemic

**DOI:** 10.1177/00207640211006153

**Published:** 2021-04-13

**Authors:** Kristina L Newman, Yadava Jeve, Pallab Majumder

**Affiliations:** 1 Psychology Department, Nottingham Trent University, UK; 2 Nottinghamshire NHS Healthcare Trust, Institute of Mental Health, University of Nottingham, UK; 3 Birmingham Women’s & Children Hospital NHS Trust, UK; 4 Nottingham City CAMHS Looked After Children’s Team, UK; 5 Division of Psychiatry & Applied Psychology, School of Medicine, University of Nottingham, UK

**Keywords:** Frontline workers, mental health, pandemic, COVID-19, coronavirus

## Abstract

**Background:**

The mental health of the population has been negatively affected due to the pandemic. Frontline healthcare workers with increased exposure to COVID diagnosis, treatment and care were especially likely to report psychological burden, fear, anxiety and depression.

**Aim:**

To elicit how working as a health professional during the pandemic is impacting on the psychological wellbeing of frontline staff.

**Method:**

United Kingdom population of healthcare workers were approached by advertising the survey via social media, NHS trusts and other organisations. Open-ended survey answers were qualitatively explored using content analysis.

**Results:**

Survey collected data from 395 NHS staff was developed into three themes; (1) Despair and uncertainty: feeling overwhelmed trying to protect everyone, (2) Behavioural and psychological impact: affecting wellbeing and functioning and (3) Coping and employer support: getting the right help.

**Conclusion:**

NHS staff felt enormous burden to adequately complete their professional, personal and civil responsibility to keep everyone safe leading to negative psychological and behavioural consequences and desire for NHS employers to offer better support. As the pandemic progresses, the results of this study may inform NHS employers on how optimum support can be offered to help them cope with negative psychological consequences of the pandemic.

## Introduction

The COVID-19 Pandemic has caused significant stress and worry in the population and frontline professionals with constant news of death rates, hospital strain and new infection rates, leading to a toll on psychological wellbeing, especially in those working on the frontline who are disproportionately exposed to the risk of transmission, morbidity and death. Research from China and other countries report that mental health of the population has been negatively affected due to the pandemic, including heightened depression, anxiety and lower overall wellbeing (Ahmed et al., 2020; Chew et al., 2020; Torales et al., 2020). Nurses and frontline healthcare workers with increased exposure to COVID diagnosis, treatment and care are especially likely to report psychological burden (Lai et al., 2020). Compared to non-clinical staff, frontline staff were 1.4 times more likely to feel fear, twice more likely to suffer anxiety and depression according to another study (Lu et al., 2020). Long work shifts with high death exposure and diverse treatment demands also added to work-related stress for staff and impacted their mental health (Neto et al., 2020). Medical staff also reported feeling anxious about being infected or passing on the condition to their families, however psychosocial protective factors included strict guidelines, availability of equipment and recognition from management and the government (Cai et al., 2020). It is, important we establish the degree and nature of psychological strain our workforce is under, while working in this pandemic and managing other responsibilities, so that this evidence can be used to optimise the support for our staff. The present study aims to elicit how working during the pandemic is impacting on the psychological wellbeing of the frontline staff in the health services, and what remedies can be put in place to help them cope with the resulting psychological difficulties.

## Method

### Design

This is a qualitative study utilising content analysis as methodology to analyse survey data.

### Participants

Any health worker within the United Kingdom was invited to take part in the survey, in order to share their experience of working on the frontline in a multitude of health service settings including primary care, community services, hospital setting, specialist or tertiary service settings and combined health-social care settings. Six hundred and twelve participants engaged with the survey, 395 completed the survey and answered a minimum of one open-ended question and were included in qualitative analysis. Table 1 shows sample demographics of the 395 who provided these details.

**Table 1.** Sample characteristics.

*N* = 395	Working on front line		Contact with COVID		Front line & contact with COVID-19
	Yes (total for now)	No	Yes	No	Yes
Gender					
Male	54	19	53	20	44
Female	247	50	223	84	205
Job					
Nursing (Reg)	121	16	108	29	99
Nursing (Non-R)	34	6	32	9	26
Doctor	72	25	71	26	61
Admin	7	9	3	13	1
Management	3	5	3	5	0
Psychology	6	4	4	6	4
Physio/OT	9	0	5	4	5
Domestic/porter	1	0	1	0	1
Midwife	26	0	24	2	24
Other	28	12	25	15	25
Age (years)					
<25	4	1	4	1	4
25–40	115	8	101	21	94
40–50	111	18	95	35	90
50–60	69	32	65	37	52
60+	10	13	10	13	5
Ethnicity					
White	225	52	200	81	180
Black	13	2	11	4	9
Mixed	6	0	5	1	4
South Asian	44	10	38	14	35
Oriental	6	1	7	0	6
Other	3	3	2	4	2

*Note*. Some missing and undisclosed information may account for numbers not fully adding up to the stated total.

### Procedure

The survey link was circulated within the health professionals working in the United Kingdom. This was advertised predominantly through social media platforms such as Twitter, WhatsApp, Facebook; and also approaching through individual local and regional organisations like NHS Trusts, Health Education bodies; and national institutes, for example, Royal Colleges of medical and allied professionals, British Fertility Society etc.

The survey was circulated for 1 month from 10th April until 10th May 2020. At the end of this duration, data was collated from responses of the completed surveys through the website of the survey. The open-ended question data acquired from the completed questionnaires was analysed with inductive qualitative content analysis (Elo & Kyngäs, 2008). Data was prepared and organised, and then coded, categorised and abstracted using NVivo12 software.

### Ethics

The authors assert that all procedures contributing to this work comply with the ethical standards of the relevant national and institutional committees on human experimentation and with the Helsinki Declaration of 1975, as revised in 2008. NHS Ethics approval was not required as this survey is not patient facing and there are no implications involving patient participation or patient information. Once developed, the survey proposal was submitted for NIHR Public Health Panel review and was institutional ethics was approved by the Governance department in Birmingham Women’s & Children Hospital NHS Trust. All participants consented by clicking and continuing with survey. Participation in the survey was voluntary and anonymous.

## Results

Using qualitative content analysis (Elo & Kyngäs, 2008), coded data was categorised and abstracted into three themes: (1) Despair and uncertainty: feeling overwhelmed trying to protect everyone, (2) Behavioural and psychological impact: affecting wellbeing and functioning and (3) Coping and employer support: getting the right help.

### Theme 1: Despair and uncertainty: Feeling overwhelmed trying to protect everyone

During this peak COVID-19 pandemic period, participants expressed a range of emotional and behavioural implications as they faced challenges such as care responsibilities, working online or redeployment. During this time, participants also had thoughts of contracting COVID-19, hopelessness, helplessness, feeling trapped, self-doubt, worthlessness, self-harm and suicidal thoughts.

Participants expressed distress in mixed messages from management, rapidly changing guidance within hours and feeling unsupported in work. Many participants were terrified of contracting COVID-19 or passing it on to vulnerable loved ones, especially if infants and young children were in the household. Some staff moved out of their family homes to try and protect their families. A minority expressed that they even regretted their choice to become a nurse.

*Anxiety from uncertainty, daily changes to processes, having to adapt my practice rapidly - a fear of ‘getting it wrong’, a fear of infecting people inadvertently, a fear of bringing home germs to my family. – P246*

*Lambs to the slaughter, risk to my family, was I going to die? Wasn’t paid enough, didn’t sign up to a death sentence – P276*

There was a deep sense of sadness for the people suffering and afraid without being able to have family or friends visit, and staff reflected on needing to have discussions about resuscitation over the phone. Staff reflected that they were saddened about not being able to care for patients as usual with ‘patients unable to see my facial expressions and unable to provide compassionate touches such as hugs or holding hands’. (P490).

For those who had clinics or departments close, such as fertility, staff despaired for their patients. Staff in intensive care and units where capacity was tight were anxious and overwhelmed. For those not on the frontline, there was a sense of guilt, whether absent due to illness, isolating due to testing positive for COVID-19, immunosuppressed or near retirement, which one participant described as feeling ‘*like a soldier missing the war*’ (P88).

*Patients faces when they are about to the induced for intubation and ventilation, they were all terrified (naturally), and I will never forget that expression of fear. Listening to some last phone or video calls between patients and family knowing they were alone. Also the sheer overwhelming feeling of being punched in the stomach the first time in ITU with double the number of patients, all extremely unstable. – P147*

New staff were ‘scared to go to work’ (P270) due to concerns of harming patients through mistakes or being asked to do something they were not comfortable with as they had limited experience. This was echoed by participants who were redeployed, who felt overwhelmed at trying to manage new roles and skills without the support of their teams and with little preparation. Many did not feel safe, feeling unprepared as they joined wards when they had not worked in that area before or had not done so for many years.

*They gave us 2 1/2 hours overview lecture on ITU setting then that is that, working in a environment that you don’t know, knowing that you might acquire the disease or worst bring it home to you family (my 2 boys are asthmatic). Asked to give medications which you’re not competent to do so, I did refuse but not received well by ITU staff. And to sum it up the FFP3 mask that I was wearing for 3 weeks was 5 years expired. My friend support worker sent me pictures. I was stressed and crying. – P270*

*Angry that there isn’t enough testing of staff and enough protection, sad that staff have died – P207*

Staff were concerned with limited or insufficient PPE due to a focus on resources rather than safety. Many staff expressed that they did not feel safe with conflicts in management, struggling to keep up with new guidance and risks of transmission by asymptomatic patients. Many staff also knew colleagues that had died or were very high risk and struggled to cope with this, feeling that their own lives were not cared about and grieving those at risk.

### Theme 2: Behavioural and psychological impact: Affecting wellbeing and functioning

Personal strain from the pandemic was also highlighted through behavioural and psychological difficulties. Changes in behaviour included reduced sleep, reduced eating, overeating, poor diet, increased irritability or anger, lack of tolerance, being distracted, making mistakes, tearfulness, self-harm, getting startled easily and avoiding going to work. While distraction and mistakes were reported at work, some other behaviour was often suppressed and emerged outside working hours such as unhealthy diets, crying in the car or heightened anxiety and irritability with family and when in public. While exhaustion and stress seemed to be at the heart of staff difficulties, they seemed to often manifest in poor diet, increased alcohol consumption and poor sleep. Some participants also expressed feeling ‘unreal’, ‘not themselves’, hearing or seeing unusual things, reoccurring dreams or nightmares, intrusive images or thoughts, flashbacks, other unusual experiences and physical symptoms (such as headaches, palpitations, sweating or shaking).

*Headaches (never normally have any headaches) stomach pains, loose stool, excessive hunger and overeating, dreaming of rooms filled with blood. Waking up anxious (never suffered with anxiety) over reliance on caffeine, going to sleep at 8pm, tired even after 10 hours sleep - P189*

Many staff reflected that these behaviours and mental health difficulties were out of the norm for them, whether as new symptoms, a relapse of difficulties not experienced in years or exacerbated existing issues. Some were surprised at their changes in mood and behaviour, saying they were ‘normally a calm, collected nurse’ (P218) or equivalent, and that ‘small things that wouldn’t normally bother me make me tearful’ (P274).

*I have suffered with lots of symptoms of anxiety, some of which I have not had in years; cold sores, trouble sleeping, fainting, crying, headaches. Extremely concerned attending work – P191*

Particularly for those redeployed, this brought exceptional strain and vulnerability where some were asked to perform duties with limited training, and concerns they were not competent in their new roles, leading to emotional distress and panic attacks. Many participants expressed doubt in their competency to fulfil their role, losing confidence in work and personal lives. A minority of participants expressed regret in their career choices as they struggled to cope in their role, planning to leave their profession post-COVID-19 for their mental health.

*The expectation to be some sort of ‘hero’ is horrible and I am not sleeping at night and having extreme panic attacks. I feel too ashamed to admit this to anyone. (. . .) The feeling that no one really cares is overwhelming. I intend to leave nursing for good after the pandemic to protect my mental health. If it survives. – P231*

Staff in more senior and managerial positions also struggled with emotional and psychological difficulties, with the added pressure of trying to support their colleagues with similar issues. The resulting distress, anxiety and panic attacks took a large toll on those affected, highlighting psychological difficulties throughout the NHS staff, regardless of role or position.

*Not feeling emotionally or psychologically strong enough to be able to support and motivate my team or support patients and their families and deal with their fears/anxieties/grief in a professional way. I have only been sleeping for around 3 hours each night due to stress and panic attacks and find myself crying almost every day as I am overwhelmed with emotion. – P277*

Outside of their work environment, participants themselves or family members also experienced relationship strain, relationships ending, domestic conflict, job loss and resulting possible financial strain, additional care responsibilities, worries about a family member or bereavement.

*I have lost much of my childcare, because usual carers are shielding [. . .] My partner is also a keyworker (full time police, shift work) and although one child has been attending school my full working hours are not covered because before and after school care has closed. Extra days at nursery will cause financial burden. – P7*

Managing childcare around shifts with no external support was difficult for parents, who also worried about the impact of home-schooling. These external stressors in addition to stressors at work and everchanging government guidelines made it difficult to cope in the pandemic, as staff felt they had no time to relax. Overall, they were clearly distressed and exhausted, and in need of support for mental and physical health. Staff albeit had an understanding that they were ‘forgetting to take care of yourself’ (P367) and to switch off from work and expressed frustration and guilt with their unhealthy behaviours and feelings of hopelessness.

### Theme 3: Coping and employer support: Getting the right help

Desired support options from employers included in the survey questionnaire were effective pastoral support, support groups, financial incentives, effective insurance cover, helplines, positive engagement from line managers, minimisation of bureaucracy and paperwork, minimise extra responsibilities, allow more time off and breaks and other techniques that many participants ticked in the quantitative section. In the qualitative responses, there was perception of significant conflict with management reported by staff who felt they were not appreciated, supported or communicated with properly. PPE shortages and redeployment to areas outside of expertise were a high point of tension with employers, with staff confused by quickly changing guidance. When staff did not feel supported by their colleagues, manager or employer, they were frustrated and distressed, with some planning to leave their profession.

*The NHS policies changing all time, there’s no coordination from management. Staff feel lost sometimes with no information and support! PPE, NOT SAFE IN MOST OF HOSPITALS! – P240*

For those that did feel supported, they were appreciative of their employers and workplaces. There was an understanding that this was also a difficult time for senior staff and managers, and across the workplace. It was acknowledged that staff were under an enormous amount of strain and that support in employment was essential through safety measures such as PPE, frequent communication, mental health and wellbeing support, management of overwork, ensuring sufficient breaks in shifts and appropriately paid overtime. They felt that an evaluation of pay was necessary to show financial recognition of their work and to reflect risk pay during the pandemic. Staff also wished for reassurance that they and their family would be taken care of financially if they fell ill working during the pandemic. Frontline staff suggested that they would like to see managers in person to support them rather than relying on emails. Those in more senior or managerial positions also highlighted that they also would like more support and communication from those above their role.

*I feel if a person is on the front line and putting their lives on the line they should be fully supported; physically with PPE, emotionally and psychology and also financially. – P470*

*Supportive attitude from the team leader can make us feel a lot more reassured, valued and worthy. It may improve our productivity and immunity by helping reduce the stress level. Very important for immediate managers (as much as the organisational leaders) are trained and reminded to do that - P24*

To help manage this high volume of psychological stress, outside of work participants pursued hobbies, exercised, meditated, cooked, completed house chores, walked or ran outside, had a support system, had a plan of action or adopted other techniques as evident from their responses to the options in the quantitative section of the survey. Participants also qualitatively described that trying to maintain a sense of normality was important, so social interaction and time outside were key points that staff felt were necessary. Keeping in contact with colleagues, friends and family by phone, social media and software such as Zoom, was greatly important for coping during the pandemic. Other distractions such as creative hobbies when feeling inspired, or entertainment through TV series and movies also helped staff to relax and enjoy themselves, however some made a conscious effort not to watch too much news which was described as ‘saddening’ and ‘horrific’.

*Exercise has been the most important thing to remove me from my thoughts and see the world outside of work and corona virus. Talking to friends and family also provides relief and support. – P97*

*I had to stop myself obsessively following all the news and statistics about Covid-19. – P439*

Finding time to get outside of the home and workplace was also important for mental health, and when managing stress, exercise was highlighted as important. For those able to exercise at home or physically distanced outside such as walking the dog, this had a beneficial effect on mental health. For those who relied on gyms or facilities such as swimming pools, this was more difficult as they felt there was ‘*nothing to let your stress out*’ (P91) due to isolation and distancing guidelines.

Some, however, felt a sense of pride for supporting their nation and the NHS in the face of the pandemic and felt that they had a sense of solidarity and support from their colleagues. This ‘awe-inspiring’ feeling may also have helped some cope with these testing times.

*Seeing my colleagues band together to cover shifts, working above and beyond their calling, leaving their families and young children at home even longer than before, bringing back the risk of contamination and death and doing it all day, everyday with that same reassuring, calming smile they reserve for their patients was awe-inspiring. – P501*

Colleagues and teams who supported each other managed the strains of the pandemic arguably more effectively than those that did not have this support network. Staff were very aware of the struggles of their colleagues, and how they felt similar fears for family and patients as they themselves did. In the face of this, there was a sense of admiration for colleagues who appeared to be coping well and assisted others. Staff wanted the best for patients, the NHS and their colleagues, but clear communication, appropriate PPE and support from senior staff and employer was felt to be essential for this.

## Discussion

This paper explores subjective experiences and perceptions of NHS staff working in the frontline during the peak of the first wave of COVID-19 pandemic in the UK in spring of 2020. Data was categorised into three themes detailing NHS frontline worker’s experiences, which were despair and uncertainty: feeling overwhelmed trying to protect everyone; behavioural and psychological impact: affecting wellbeing and functioning; and coping and employer support: getting the right help. Staff clearly highlighted that they are struggling with their work pressure, feelings of uncertainty and risk at work and in general and that the pandemic is taking a significant toll on their psychological wellbeing.

Media has also highlighted the negative effects on mental health, with the continuing escalation of the pandemic increasing stress related difficulties, in addition to exacerbating existing mental health conditions (Shuja et al., 2020). The need for mental health support for those infected and vulnerable has also been highlighted by both Orru et al. (2020) and Duan and Zhu (2020). Due to strict infection control measures and distancing guidelines, mental health support staff may often be not available or only remotely accessible on the physical health wards, resulting in psychological support being provided primarily by available frontline workers. From this research and others, we see that frontline workers are already exhausted and struggling with balancing their own physical and mental health (Greenberg et al., 2020), and are in need of support themselves rather than being assigned with further responsibilities. This was also recommended by Ho et al. (2020), who suggest that support from mental health professionals such as psychologists and psychiatrists to patients would increase efficacy of interventions to support mental health and reduce strain on frontline staff.

China has since called for an emergency psychological crisis intervention with mental health video contents and online mental health support for both health professionals and the public (Li et al., 2020) in response to the overwhelming negative impact on overall mental health due to the pandemic. Wang et al. (2020a) found that lower levels of stress, depression and anxiety were associated with health information (e.g. treatment availability, local outbreak situation), perceived low risk of contracting COVID-19 and survival likelihood, high confidence in doctors, and personal precautionary measures (e.g. washing hands, wearing a mask). Dissemination of unbiased COVID-19 knowledge, financial support and availability of essential services and commodities should therefore be the priority of governments looking to support population mental health (Wang et al., 2020b). More specifically for health professionals, early support, anticipating and supporting with possible moral injury (making difficult decisions against moral judgement), honest discussions on what staff will face, regular contact to discuss decisions and wellbeing, and aftercare from management post-pandemic (Greenberg et al., 2020) appears to be essential strategies for employers to adopt. These are consistent with the findings of this study where the frontline NHS workers shared their desire to have similar support mechanisms at their disposal.

### Strengths and limitations

This study boasts a substantial sample from a range of NHS roles and demographic representations. Participants readily provided honest accounts of their thoughts and feelings, leading to rich data for analysis. The despair and helplessness expressed by the participants give a clear indication of their struggles. To our knowledge this survey is the earliest in the country to be conducted with the health workers, that was run during the period building up to the first peak of the pandemic. Therefore, the responses are less likely to have memory bias and would capture an accurate account of the staff experience in real time. The circulation of the survey predominantly through more informal means such as social media is more likely to generate honest and genuine response from staff about their emotional struggles and dissatisfaction with the employer, as compared to surveys circulated by employers themselves that might restrict accurate reflection due to fear of repercussions.

The limitation of this study is the restriction of the nature of survey questions, with limited room for detailed answers, probing questions, opportunity to seek clarification or further information that would have been possible if other tools and methodologies were adopted, such as semi-structured interviews. Furthermore, the open-ended questions were not compulsory, and so some participants did not complete them.

### Implications and applications

This study provides a unique perspective of NHS staff at the peak of the COVID-19 pandemic in May 2020, before we understood the pandemic, had management and PPE and before vaccines were in development. The data highlights the distress and uncertainty experienced by staff as they felt enormous burden to adequately complete their professional, personal and civil responsibility to keep everyone safe leading to negative psychological and behavioural consequences and desire to receive a better support from the NHS employers. The results of this study may inform NHS employers and management staff on areas of employee mental health which need further support as we progress through the subsequent phases of the pandemic, and into long-term recovery of COVID-19. A follow-up qualitative study may focus on interpretative phenomenological analysis (IPA) to further unpack NHS staff experiences of COVID-19. A further longitudinal study to look at the experiences of NHS staff at managing COVID-19 during the ‘second wave’ and the effects of ‘long-term’ COVID symptoms may also be useful to develop strategies and operationalise optimum NHS staff support.

## Conclusion

This study provides a unique perspective of NHS staff at the peak of the COVID-19 pandemic in May 2020, before we understood the pandemic, had management and PPE, and before vaccines were in development. The data highlights the distress and uncertainty experienced by staff as they felt enormous burden to adequately complete their professional, personal and civil responsibility to keep everyone safe leading to negative psychological and behavioural consequences and desire to receive a better support from the NHS employers. The results of this study may inform NHS employers on areas of employee mental health which needs further support as we progress through potential second waves, and into long-term recovery of COVID-19.

## Supplemental Material

sj-pdf-1-isp-10.1177_00207640211006153 – Supplemental material for Experiences and emotional strain of NHS frontline workers during the peak of the COVID-19 pandemic Click here for additional data file. Supplemental material, sj-pdf-1-isp-10.1177_00207640211006153 for Experiences and emotional strain of NHS frontline workers during the peak of the COVID-19 pandemic by Kristina L Newman, Yadava Jeve and Pallab Majumder in International Journal of Social Psychiatry
