# Supplementary material for: Experiences and emotional strain of NHS frontline workers during the peak of the COVID-19 pandemic
Source: Int J Soc Psychiatry. 2021 Apr 13;68(4):783–90. doi: 10.1177/00207640211006153 (PMC9014765; doi:10.1177/00207640211006153)
Supplement: sj-pdf-1-isp-10.1177_00207640211006153 – Supplemental material for Experiences and emotional strain of NHS frontline workers during the peak of the COVID-19 pandemic [file sj-pdf-1-isp-10.1177_00207640211006153.pdf]

Survey: Experience of frontline health staff of working in the COVID19 Pandemic

Q1. Have you worked on the frontline during the COVID19 Pandemic?

Yes No

Q2. Did you come in direct contact with suspected or confirmed cases of COVID19?

Yes No

Q3. How stressful did you find working during this time?

None Only little Moderate Quite a lot Extremely

Q4. What were the significant negative feelings you mostly experienced during this time (tick one or more)?

Anger

Frustration

Sadness

Fear

Anxiety

Unsafe

Q5. Please can you expand on the above responses?

Q6. What were the significant positive feelings you mostly experienced during this time (tick one or more)?

Gratitude

Calmness

Solidarity

Peace

Happiness

Hope

Compassion

Sense of purpose

Q7. Please can you expand on the above responses?

Q8. Have you noticed any of the following changes in your behaviour during this time (tick one or more)?

Reduced sleep

Reduced eating

Overeating

Increased irritability/ anger

Lack of tolerance

Lashing out

Being distracted

Making mistakes

Tearfulness

Self-harm

Getting startled easily

Avoiding going to work

Q9. Please can you expand on the above responses?

Q10. Have you noticed any of the following thoughts during this time (tick one or more)?

Contracting covid19

Hopelessness

Helplessness

Trapped

Self-doubt

Worthlessness

Self-harm thoughts

Suicidal thoughts

Q11. Please can you expand on the above responses?

Q12. Have you had any of the following experiences during this time (tick one or more)?

Feeling unreal

Feeling not yourself

Hearing unusual things

Seeing unusual things

Repeated dreams/ nightmares

Intrusive images/ thoughts

Flashbacks

Any other unusual experience

Any physical symptoms (headache, palpitation, sweating, shaking)

Q13. Please can you expand on the above responses?

Q14. Have you or any family members experienced any of the following (tick one or more)?

Relationship strain

Break up of relationship

Domestic conflict

Job loss

Financial strain

Additional care responsibility

Worry about a family member

Bereavement

Q15. Please can you expand on the above responses?

Q16. Which of the following strategies have you found helpful in managing psychological stress during this time (tick one or more)?

Pursuing hobbies

Exercise

Meditation

Cooking

House chores

Walk/ run outside

Presence of support system

Having a plan of action

Other

Q17. Please can you expand on the above responses?

Q18. How much do you think your strategies are helpful on a scale from 0 – 10 (please put a number)?

Q19. What do you think your employer should do to support you in the best possible way (tick one or more)?

Effective Pastoral support

Support group

Financial incentive

Effective insurance cover

Helpline to talk

Positive engagement from line manger

Minimise bureaucracy/ paperwork

Minimise extra responsibilities

Allow more time off/ breaks

Other

Q20. Please can you expand on the above responses?
